# A multi‐country cross‐sectional study to assess predictors of daily versus on‐demand oral pre‐exposure prophylaxis in youth from South Africa, Uganda and Zimbabwe

**DOI:** 10.1002/jia2.25975

**Published:** 2022-08-24

**Authors:** Janan Janine Dietrich, Nadia Ahmed, Emily L. Webb, Gugulethu Tshabalala, Stefanie Hornschuh, Mamakiri Mulaudzi, Millicent Atujuna, Lynda Stranix‐Chibanda, Teacler Nematadzira, Andrew Sentoogo Ssemata, Richard Muhumuza, Janet Seeley, Linda‐Gail Bekker, Helen A. Weiss, Neil Martinson, Julie Fox, the CHAPS team

**Affiliations:** ^1^ Perinatal HIV Research Unit (PHRU) Faculty of Health Sciences University of the Witwatersrand Johannesburg South Africa; ^2^ Health Systems Research Unit, South African Medical Research Council, Bellville South Africa and African Social Sciences Unit of Research and Evaluation (ASSURE) Wits Health Consortium University of the Witwatersrand Johannesburg South Africa; ^3^ Mortimer Market Centre Central North West London NHS Trust London United Kingdom; ^4^ MRC International Statistics and Epidemiology Group London School of Hygiene and Tropical Medicine London United Kingdom; ^5^ Desmond Tutu HIV Centre University of Cape Town Cape Town South Africa; ^6^ Clinical Trials Research Centre University of Zimbabwe Harare Zimbabwe; ^7^ Child and Adolescent Health Unit Faculty of Medicine and Health Sciences University of Zimbabwe Harare Zimbabwe; ^8^ Medical Research Council/Uganda Virus Research Institute and London School of Hygiene & Tropical Medicine Uganda Research Unit Entebbe Uganda; ^9^ Global Health and Development Department London School of Hygiene and Tropical Medicine London United Kingdom; ^10^ Guys and St Thomas’ NHS Trust/King's College London London United Kingdom

**Keywords:** Africa, HIV, on‐demand, oral pre‐exposure prophylaxis, young people

## Abstract

**Introduction:**

Sub‐Saharan Africa (SSA) carries the burden of the HIV epidemic, especially among adolescents and young people (AYP). Little is known about pre‐exposure prophylaxis (PrEP) uptake and preferences among AYP in SSA. We describe preferences for daily and on‐demand PrEP among AYP in South Africa, Uganda and Zimbabwe.

**Methods:**

A cross‐sectional survey was conducted in 2019 among 13‐ to 24‐year olds, capturing socio‐demographics, HIV risk behaviours and preferences for daily or on‐demand PrEP. Logistic regression models were used to estimate odds ratios, adjusting for site, sex and age.

**Results and discussion:**

A total of 1330 participants from Cape Town (*n* = 239), Johannesburg (*n* = 200), Entebbe (*n* = 491) and Chitungwiza (*n* = 400) were enrolled; 673 (51%) were male, and the median age was 19 years (interquartile range 17–22 years). Of 1287 participants expressing a preference, 60% indicated a preference for on‐demand PrEP with differences by site (*p* < 0.001), sex (*p* < 0.001) and age group (*p* = 0.003). On‐demand PrEP was most preferred in Entebbe (75%), among males (65%) versus females (54%) and in older participants (62% in 18‐ to 24‐year‐olds vs. 47% in 13‐ to 15‐year‐olds). After adjusting for site, sex and age group, preference for on‐demand PrEP decreased as sex frequency over the past month increased (*p*‐trend = 0.004) and varied with the number of partners in the last 6 months, being least popular among those reporting four or more partners (*p* = 0.02). Participants knowing further in advance that they were likely to have sex were more likely to prefer on‐demand PrEP (*p*‐trend = 0.02). Participants having a larger age gap with their most recent partner and participants whose last partner was a transactional sex partner or client were both less likely to prefer on‐demand compared to daily PrEP (*p* = 0.05 and *p* = 0.09, respectively). Participants who knew their most recent partner was living with HIV or who did not know the HIV status of their most recent partner were less likely to prefer on‐demand PrEP (*p* = 0.05).

**Conclusions:**

Our data show that AYP in four SSA communities prefer on‐demand over daily PrEP options, with differences seen by site, age and sex. PrEP demand creation needs to be reviewed, optimized and tailored to socio‐demographic differences and designed in conjunction with AYP.

## 1 INTRODUCTION

Sub‐Saharan Africa (SSA) is home to 89% of the 1.75 million adolescents 10–19 years living with HIV worldwide, with approximately 150,000 new infections among this age group in 2020 [1, 2]. Given the social determinants of health in SSA, adolescents and young people (AYP) remain vulnerable to acquiring HIV through sexual transmission [3, 4].

Antiretroviral treatment prevents HIV acquisition through pre‐ and post‐exposure prophylaxis (PrEP and PEP, respectively). PrEP shows the efficacy of 86% with high adherence [5, 6], and is recommended for those at substantial risk of acquiring HIV. An on‐demand regimen, known as PrEP 2‐1‐1, is effective in men having sex with men (MSM) [7]. The on‐demand dosing is two pills 2–24 hours before sexual activity, one pill 24 hours after the first dose and one pill 24 hours after the second dose [7].

Currently, more than 100 countries have PrEP guidelines, with varying degrees of implementation [8, 9]. South Africa was the first country in SSA to rollout PrEP in June 2016 [10] with PrEP offered in 2018, to sexually active, HIV‐negative female AYP [10]. Uganda followed in November 2016 with PrEP available only through demonstration facilities [11]. Zimbabwe introduced PrEP in May 2016, in the private sector and demonstration projects for adolescent girls and young women [12].

A few trials included adolescents below 18 years [13], but those in young adults showed that less than one‐third had evidence of taking PrEP through plasma drug levels [14, 15]. The effectiveness of peer support and mobile technology on adherence is being investigated [16, 17], as well as trials for different delivery mechanisms and biomedical modalities [18]. Despite ongoing efforts, AYP continue to be vulnerable to HIV [19].

The Combined HIV Adolescent PrEP and Prevention Study (CHAPS) was a mixed‐methods study investigating daily and on‐demand PrEP among AYP in SSA [18]. Although on‐demand PrEP is presently recommended only for MSM [20], studies were conducted among adult key populations [21, 22], with a lack of research among heterosexual AYP [23, 24]. We investigated preferences for daily and on‐demand PrEP and its predictors, among AYP in South Africa, Uganda and Zimbabwe.

## 2 METHODS

### 2.1 Study sites

We conducted cross‐sectional surveys, between May and December 2019, at four sites in South Africa, Uganda and Zimbabwe. At this time, PrEP was available in South Africa but not in Uganda and Zimbabwe. There is a lack of data around PrEP uptake and preferences in all three countries among AYP, who contribute to the global HIV incidence [7, 14, 25, 26].

### 2.2 Participant sampling and procedures

Trained fieldworkers used a purposive community outreach strategy to recruit participants in highly populous informal peri‐urban communities, including informal settlements and areas with low‐cost government housing [27]. Participants were from comparable communities characterized by high unemployment, low household incomes, overcrowding, limited resources and service delivery [28].

In Zimbabwe and South Africa, participants were recruited in locations where young people meet. In Uganda, participants were approached in fishing communities through local leaders, project mobilizers and village health teams. We aimed to survey a target of 400 participants in each country stratified by age (13–15, 16–17 and 18–24 years in 1:2:4 ratio) and gender (male and female in 1:1 ratio). As the main study was descriptive, no formal sample size calculation was performed.

Eligible participants were 13–24 years, self‐reported sex in the past 6 months at screening (South Africa and Zimbabwe only) and were willing to undergo rapid HIV testing to confirm HIV status. Participants with a confirmed positive HIV test were supported and referred to healthcare facilities for care. Participants who were deemed eligible and tested HIV negative were enrolled.

### 2.3 Data collection procedures

Using Open Data Kit [29], fieldworkers administered a structured survey (available in English and local languages) using computer tablets. A description of daily and on‐demand PrEP was provided to ensure understanding about the choices in the survey. Following consent/assent procedures, participants responded to the interviewer‐administered survey in a confidential and convenient location.

## 3 MEASURES

### 3.1 Outcome variable

The main outcome was PrEP preference, assessed by: “At the moment, do you think you would prefer on‐demand or daily PrEP?” with response options on‐demand, daily, unsure and no preference. We also asked about PrEP‐related attitudes, including whether participants had heard of PrEP, would use PrEP, main reasons for preferring on‐demand PrEP or daily PrEP, respectively.

### 3.2 Exposure variables

Table 1 lists exposure variables: socio‐demographics, sexual risk behaviour and PrEP‐related disclosure.

**Table 1:** Distribution of overall and site characteristics of AYP participating in the CHAPS survey

Characteristic	Category	Cape Town (*n* = 239)	Johannesburg (*n* = 200)	Entebbe (*n* = 491)	Chitungwiza (*n* = 400)	Total (*n* = 1330)
Sex	Male	124 (52%)	99 (50%)	250 (51%)	200 (50%)	673 (51%)
	Female	115 (48%)	101 (51%)	241 (49%)	200 (50%)	657 (49%)
Age group, years	13–15	37 (15%)	21 (11%)	52 (11%)	40 (10%)	150 (11%)
	16–17	44 (18%)	33 (17%)	83 (17%)	80 (20%)	240 (18%)
	18–24	158 (66%)	146 (73%)	356 (73%)	280 (70%)	940 (71%)
Highest education	Still studying	141 (59%)	126 (63%)	226 (46%)	203 (51%)	696 (52%)
	<Grade 7	1 (0.4%)	0 (0%)	118 (24%)	9 (2.4%)	128 (9.6%)
	Grade 7–12	87 (36%)	67 (34%)	136 (28%)	169 (42%)	459 (35%)
	Post‐school	10 (4.2%)	7 (4%)	11 (2.2%)	19 (4.8%)	47 (3.5%)
Sex frequency, past month^a^	At least daily	18 (8.4%)	11 (5.9%)	8 (1.7%)	15 (3.8%)	52 (4.1%)
	2–3 times/week	63 (29%)	56 (30%)	59 (12%)	56 (14%)	234 (18%)
	Once/week	68 (32%)	41 (22%)	52 (11%)	56 (14%)	217 (17%)
	Once/month	41 (19%)	52 (27%)	61 (13%)	71 (18%)	224 (18%)
	Never	24 (11%)	27 (15%)	294 (62%)	202 (51%)	547 (43%)
Advanced knowledge of last sexual encounter	<2 hours	120 (50%)	96 (48%)	123 (37%)	142 (51%)	481 (46%)
	2–12 hours	58 (24%)	51 (26%)	54 (16%)	45 (16%)	208 (20%)
	13–24 hours	15 (6.3%)	28 (14%)	31 (9.3%)	17 (6.2%)	91 (8.7%)
	>24 hours	45 (19%)	23 (12%)	127 (38%)	72 (26%)	267 (26%)
Number of partners, last 6 months^a^	0	5 (2.2%)	0 (0%)	206 (42%)	153 (38%)	364 (28%)
	1	115 (50%)	79 (41%)	192 (39%)	130 (33%)	516 (39%)
	2	59 (25%)	51 (26%)	47 (9.6%)	53 (13%)	210 (16%)
	3	32 (14%)	34 (17%)	18 (3.7%)	33 (8.3%)	117 (8.9%)
	4 or more	21 (9.1%)	31 (16%)	28 (5.7%)	31 (7.8%)	111 (8.4%)
Age of most recent partner^a^	>5 years younger	2 (0.9%)	1 (0.5%)	1 (0.3%)	10 (3.7%)	14 (1.4%)
	1–5 years younger	65 (28%)	52 (27%)	124 (38%)	70 (26%)	311 (30%)
	Same age	72 (31%)	44 (23%)	33 (10%)	61 (22%)	210 (20%)
	1–5 years older	72 (31%)	73 (37%)	120 (37%)	87 (32%)	352 (34%)
	>5 years older	21 (9.1%)	25 (13%)	48 (15%)	44 (16%)	138 (13%)
Relationship with most recent partner^a^	Regular partner	197 (83%)	161 (81%)	297 (89%)	208 (75%)	863 (83%)
	Casual partner	40 (17%)	37 (19%)	37 (11%)	63 (23%)	177 (17%)
	Transactional sex	1 (0.4%)	0 (0%)	0 (0%)	5 (1.8%)	6 (0.6%)
HIV status of most recent partner^a^	Positive	3 (1.3%)	0 (0%)	2 (0.6%)	1 (0.4%)	6 (0.6%)
	Negative	134 (57%)	101 (51%)	192 (57%)	176 (64%)	603 (58%)
	Don't know	98 (42%)	97 (49%)	141 (42%)	99 (36%)	435 (42%)
Perceived change of acquiring HIV in next 3 months	No chance	114 (48%)	108 (54%)	359 (73%)	269 (67%)	850 (64%
	Some chance	90 (38%)	67 (34%)	108 (22%)	101 (25%)	366 (28%)
	Moderate change	28 (12%)	17 (8.5%)	20 (4.1%)	23 (5.8%)	88 (6.6%)
	High chance	7 (2.9%)	8 (4.0%)	4 (0.8%)	7 (1.8%)	26 (2.0%)
Had heard of PrEP^a^	No	125 (53%)	128 (64%)	432 (88%)	309 (77%)	994 (75%)
	Yes	113 (47%)	72 (36%)	59 (12%)	91 (23%)	335 (25%)
Would disclose PrEP use to partner	No	63 (29%)	58 (30%)	144 (31%)	165 (42%)	430 (34%)
	Yes	157 (71%)	137 (70%)	325 (69%)	228 (58%)	847 (66%)

^a^ Missing values for these variables.

### 3.3 Ethical considerations

Study procedures were approved per country requirements. Written informed consent was obtained from participant ≥18 years. Parental consent and participant assent were obtained for participants ≤17 years. Parental waivers were in place in Uganda, Zimbabwe and Cape Town. Participants were reimbursed for time and participation according per country requirements. To limit potential stigma, study sites collaborated with local community advisory boards.

### 3.4 Data analysis

Data were analysed in Stata version 15 (StataCorp, Texas, USA) [30]. Participants indicating preferences for daily/on‐demand PrEP were included for analysis. The outcome was PrEP preference: daily versus on‐demand. Descriptive statistics summarized the number and proportion of participants expressing a preference for daily versus on‐demand PrEP. Logistic regression models were fitted to generate crude and adjusted odds ratios (aOR)—adjusted for site, sex and age—and 95% confidence intervals (CI) for association between each exposure variable and the outcome, using daily PrEP as the reference group; *p*‐values were calculated from likelihood ratio tests. Tests for trend were conducted for ordered categorical exposures. Effect modification by site and sex was investigated using likelihood ratio tests.

## 4 RESULTS AND DISCUSSION

### 4.1 PrEP characteristics

A total of 1330 participants from Cape Town (*n* = 239), Johannesburg (*n* = 200), Entebbe (*n* = 491) and Chitungwiza (*n* = 400) participated in the survey; 673 (51%) were male, the median age was 19 years (interquartile range 17–22 years) and 699 (52%) were still studying. Of these, 43 stated that they had no preference for either daily or on‐demand PrEP. Of the remaining 1287 participants expressing a PrEP preference, 25% had heard of PrEP prior to taking the survey, 95% said that they would use PrEP and more than half (60%) preferred on‐demand to daily PrEP. In crude analysis, PrEP preference varied by site (*p* < 0.001), sex (*p* < 0.001) and age group (*p* = 0.003). On‐demand PrEP was most popular in Entebbe (75%) and least popular in Cape Town (32%) (*p* < 0.001), more popular among males than females (65% vs. 54%; *p* < 0.001) and more popular in 18‐ to 24‐year‐olds than 16‐ to 17‐ or 13‐ to 15‐year‐olds (62%; 57%; and 47%; *p*‐trend = 0.001).

Preference for on‐demand PrEP was associated with lower‐risk behaviours (Table 2). Preference for on‐demand PrEP decreased as sex frequency over the past month increased (*p*‐trend = 0.004) and varied with the number of recent partners, being least popular among those reporting four or more partners (*p* = 0.02). Participants who knew further in advance that they were likely to have sex were more likely to prefer on‐demand PrEP (*p*‐trend = 0.02). Participants who had a larger age gap with their most recent partner and participants whose last partner was a transactional sex partner were both less likely to prefer on‐demand PrEP (*p* = 0.05 and *p* = 0.09, respectively). Participants who knew that their most recent partner was living with HIV or who did not know the HIV status of their most recent partner were also less likely to prefer on‐demand PrEP (*p* = 0.05).

**Table 2:** Factors associated with preference for on‐demand versus daily PrEP, after adjustment for site, sex and age group

Characteristic	Category	Prefer daily	Prefer on‐demand	Total	Crude OR (95% CI)	*p*‐value	Adjusted OR (95% CI)	*p*‐value
Site	Cape Town	146 (68%)	68 (32%)	214	0.16 (0.11, 0.22)	<0.001	0.15 (0.11, 0.22)	<0.001
	Johannesburg	84 (46%)	100 (54%)	184	0.40 (0.28, 0.57)		0.39 (0.28, 0.56)	
	Entebbe	124 (25%)	367 (75%)	491	Baseline		Baseline	
	Zimbabwe	167 (42%)	231 (58%)	398	0.47 (0.35, 0.62)		0.46 (0.35, 0.62)	
Sex	Male	225 (35%)	422 (65%)	647	Baseline	<0.001	Baseline	<0.001
	Female	296 (46%)	344 (54%)	640	0.62 (0.50, 0.78)		0.59 (0.47, 0.75)	
Age group	13–15	74 (53%)	65 (47%)	139	0.54 (0.38, 0.77)	0.003	0.53 (0.36, 0.78)	0.004
	16–17	98 (43%)	132 (57%)	230	0.83 (0.62, 1.11)	0.001 (trend)	0.85 (0.62, 1.15)	0.001 (trend)
	18–24	349 (38%)	569 (62%)	918	Baseline		Baseline	
No. of partners, last 6 months	0	141 (39%)	222 (61%)	363	1.06 (0.81, 1.40)	0.41	0.69 (0.49, 0.97)	0.02
	1	200 (40%)	296 (60%)	496	Baseline	0.20 (trend)	Baseline	0.85 (trend)
	2	73 (37%)	126 (63%)	199	1.17 (0.83, 1.64)		1.26 (0.87, 1.84)	
	3	50 (46%)	59 (54%)	109	0.80 (0.53, 1.21)		0.81 (0.51, 1.29)	
	4 or more	49 (45%)	59 (54%)	108	0.81 (0.54, 1.24)		0.65 (0.41, 1.03)	
Sex frequency past month	At least daily	29 (59%)	20 (41%)	49	0.35 (0.20, 0.64)	<0.001	0.55 (0.29, 1.06)	0.24
	2–3 times a week	108 (47%)	120 (53%)	228	0.57 (0.42, 0.78)	<0.001 (trend)	0.72 (0.50, 1.05)	0.04 (trend)
	Once a week	89 (43%)	117 (57%)	206	0.68 (0.49, 0.94)		0.95 (0.64, 1.39)	
	Once a month	87 (41%)	124 (59%)	211	0.73 (0.53, 1.02)		0.95 (0.66, 1.36)	
	Never	184 (34%)	358 (66%)	542	Baseline		Baseline	
Last time had sex, how far in advance knew	<2 hours	209 (46%)	246 (54%)	455	Baseline	<0.001	Baseline	0.07
	2–12 hours	83 (41%)	121 (59%)	204	1.24 (0.89, 1.73)	<0.001 (trend)	1.35 (0.94, 1.93)	0.02 (trend)
	13–24 hours	34 (39%)	53 (61%)	87	1.32 (0.83, 2.12)		1.09 (0.66, 1.81)	
	>24 hours	76 (29%)	183 (71%)	259	2.05 (1.48, 2.83)		1.56 (1.09, 2.22)	
Age most recent partner	>5 years younger	9 (64%)	5 (36%)	14	0.48 (0.15, 1.47)	<0.001	0.34 (0.10, 1.10)	0.05
	1–5 years younger	91 (31%)	205 (69%)	296	1.93 (1.33, 2.81)		1.25 (0.83, 1.90)	
	Same age	91 (46%)	106 (54%)	197	Baseline		Baseline	
	1–5 years older	135 (40%)	206 (60%)	341	1.31 (0.92, 1.87)		1.40 (0.90, 2.17)	
	>5 years older	66 (48%)	71 (52%)	137	0.92 (0.60, 1.43)		0.94 (0.655 1.61)	
Relationship, last partner	Regular partner	336 (41%)	494 (60%)	830	Baseline	0.11	Baseline	0.09
	Casual partner	61 (36%)	107 (64%)	168	1.19 (0.85, 1.68)		1.11 (0.76, 1.62)	
	Transactional sex	5 (83%)	1 (17%)	6	0.14 (0.02, 1.17)		0.13 (0.01, 1.18)	
HIV status recent partner	Negative	227 (39%)	357 (61%)	584	Baseline	0.15	Baseline	0.05
	Positive	4 (80%)	1 (20%)	5	0.16 (0.02, 1.43)		0.16 (0.02, 1.58)	
	Don't know	169 (41%)	244 (59%)	413	0.92 (0.71, 1.19)		0.77 (0.57, 1.02)	
Perceived chance of HIV next 3 months	No chance	299 (36%)	523 (64%)	822	Baseline	<0.001	Baseline	0.06
	Some chance	160 (45%)	194 (55%)	354	0.69 (0.54, 0.89)	<0.001 (trend)	0.77 (0.58, 1.01)	0.006 (trend)
	Moderate chance	47 (55%)	39 (45%)	86	0.47 (0.30, 0.74)		0.64 (0.39, 1.03)	
	High chance	15 (60%)	10 (40%)	25	0.38 (0.17, 0.86)		0.50 (0.21, 1.20)	
Would disclose PrEP to partner	No	148 (36%)	266 (64%)	414	Baseline	0.03	Baseline	0.01
	Yes	348 (42%)	477 (58%)	825	0.76 (0.60, 0.97)		0.71 (0.55, 0.92)	

Participants perceiving a higher chance of acquiring HIV in the next 3 months and participants willing to disclose their PrEP usage to a partner were less likely to prefer on‐demand PrEP (*p*‐trend = 0.006, *p* = 0.01, respectively). There was no evidence of association with PrEP preference for any of the other exposures examined. Regarding effect modification, there was some suggestion that the association of age group with PrEP preference differed by site (Table 3). Younger participants in Cape Town were more likely to prefer on‐demand PrEP, while older participants from the other three sites were more likely to prefer daily PrEP. There was little evidence of effect modification by site or sex for any of the other associations seen.

**Table 3:** Adjusted associations between participant characteristics and preference for on‐demand versus daily PrEP, overall and separately for each CHAPS survey setting

		Overall, all settings (*n* = 1287)	Cape Town (*n* = 214)	Johannesburg (*n* = 184)	Uganda (*n* = 491)	Zimbabwe (*n* = 398)	
Characteristic	Category	Adjusted OR (95% CI)	Adjusted OR (95% CI)	Adjusted OR (95% CI)	Adjusted OR (95% CI)	Adjusted OR (95% CI)	Interaction *p*‐value^a^
Sex	Male	Baseline	Baseline	Baseline	Baseline	Baseline	0.02
	Female	0.59 (0.47, 0.75)	0.82 (0.46, 1.46)	0.67 (0.37, 1.21)	0.80 (0.53, 1.22)	0.34 (0.23, 0.52)	
Age group	13–15	0.53 (0.36, 0.78)	1.86 (0.81, 4.23)	0.71 (0.26, 1.91)	0.37 (0.20, 0.68)	0.32 (0.16, 0.66)	0.01
	16–17	0.85 (0.62, 1.15)	1.79 (0.86, 3.72)	0.57 (0.25, 1.30)	0.59 (0.35, 1.01)	0.93 (0.55, 1.57)	
	18–24	Baseline	Baseline	Baseline	Baseline	Baseline	
Age of first sex	Per unit increase	1.02 (0.96, 1.09)	1.04 (0.89, 1.23)	0.93 (0.79, 1.09)	1.06 (0.96, 1.17)	1.02 (0.88, 1.18)	0.55
Transactional sex, ever	No	Baseline	Baseline	Baseline	Baseline	Baseline	0.14
	Yes	0.92 (0.61, 1.38)	0.46 (0.13, 1.69)	1.75 (0.62, 4.97)	0.77 (0.38, 1.53)	0.99 (0.47, 2.07)	
Forced sex, last 6 months	No	Baseline	Baseline	Baseline	Baseline	Baseline	0.57
	Yes	1.10 (0.65, 1.87)	0.85 (0.21, 3.51)	0.86 (0.21, 3.59)	1.44 (0.61, 3.38)	0.65 (0.23, 1.84)	
Forced someone to have sex, last 6 months	No	Baseline	Baseline	Baseline	Baseline	Baseline	0.74
	Yes	0.98 (0.52, 1.85)	0.53 (0.10, 2.70)	1.08 (0.23, 5.05)	1.53 (0.43, 5.42)	0.75 (0.23, 2.44)	
No. of partners, last 6 months	0	0.69 (0.49, 0.97)	1.56 (0.24, 9.95)	–	0.78 (0.45, 1.33)	0.74 (0.42, 1.30)	0.70
	1	Baseline	Baseline	Baseline	Baseline	Baseline	
	2	1.26 (0.87, 1.84)	1.64 (0.78, 3.44)	1.61 (0.74, 3.52)	1.23 (0.53, 2.85)	0.96 (0.47, 1.99)	
	3	0.81 (0.51, 1.29)	1.03 (0.38, 2.76)	1.31 (0.53, 3.24)	1.16 (0.31, 4.34)	0.46 (0.20, 1.03)	
	4 or more	0.65 (0.41, 1.03)	0.60 (0.17, 2.16)	1.09 (0.43, 2.80)	0.40 (0.16, 0.96)	0.83 (0.34, 1.99)	
Sex frequency past month	At least daily	0.55 (0.29, 1.06)	0.21 (0.05, 0.97)	0.54 (0.11, 2.63)	0.44 (0.10, 1.93)	0.50 (0.16, 1.55)	0.70
	2–3 times a week	0.72 (0.50, 1.05)	0.40 (0.14, 1.13)	0.35 (0.12, 1.05)	0.76 (0.38, 1.52)	0.76 (0.40, 1.47)	
	Once a week	0.95 (0.64, 1.39)	0.48 (0.18, 1.33)	0.40 (0.13, 1.25)	1.00 (0.47, 2.13)	1.01 (0.52, 1.98)	
	Once a month	0.95 (0.66, 1.36)	0.37 (0.12, 1.14)	0.38 (0.13, 1.15)	1.22 (0.58, 2.55)	0.88 (0.48, 1.61)	
	Never	Baseline	Baseline	Baseline	Baseline	Baseline	
Last time had sex, how far in advance knew	<2 hours	Baseline	Baseline	Baseline	Baseline	Baseline	0.74
	2–12 hours	1.35 (0.94, 1.93)	1.72 (0.84, 3.52)	1.26 (0.62, 2.58)	0.89 (0.43, 1.87)	1.70 (0.80, 3.63)	
	13–24 hours	1.09 (0.66, 1.81)	0.40 (0.08, 1.94)	0.97 (0.40, 2.36)	1.92 (0.61, 6.00)	0.96 (0.32, 2.88)	
	>24 hours	1.56 (1.09, 2.22)	1.64 (0.74, 3.63)	1.16 (0.42, 3.22)	1.37 (0.74, 2.53)	1.87 (0.96, 3.62)	
Current relationship status	Single	1.04 (0.71, 1.54)	2.16 (0.98, 4.76)	1.25 (0.51, 3.02)	0.75 (0.36, 1.56)	0.72 (0.35, 1.49)	0.61
	Boyfriend/girlfriend	Baseline	Baseline	Baseline	Baseline	Baseline	
	Other	0.94 (0.60, 1.48)	1.35 (0.32, 5.70)	0.91 (0.05, 15.06)	0.83 (0.43, 1.58)	1.20 (0.55, 2.62)	
Age gap, last partner	Same age	Baseline	Baseline	Baseline	Baseline	Baseline	0.78
	1–5 years gap	1.30 (0.90, 1.87)	1.38 (0.70, 2.70)	1.02 (0.47, 2.21)	1.95 (0.85, 4.50)	1.38 (0.67, 2.85)	
	>5 years gap	0.80 (0.49, 1.33)	1.17 (0.37, 3.64)	0.39 (0.12, 1.20)	1.12 (0.39, 3.24)	1.06 (0.43, 2.61)	
Relationship, last partner	Regular sexual partner	Baseline	Baseline	Baseline	Baseline	Baseline	0.09
	Other	1.03 (0.71, 1.50)	1.15 (0.52, 2.56)	0.65 (0.30, 1.44)	0.74 (0.32, 1.68)	1.59 (0.82, 3.05)	
Condom use, last sex	No	Baseline	Baseline	Baseline	Baseline	Baseline	0.45
	Yes	0.87 (0.66, 1.15)	0.92 (0.50, 1.70)	0.62 (0.33, 1.15)	0.96 (0.55, 1.70)	0.90 (0.54, 1.53)	
HIV status, last partner	Negative	Baseline	Baseline	Baseline	Baseline	Baseline	0.29
	Positive/don't know	0.75 (0.56, 1.00)	0.84 (0.45, 1.57)	0.59 (0.31, 1.13)	0.72 (0.42, 1.23)	0.91 (0.52, 1.59)	
Condom use past 6 months	Never	Baseline	Baseline	Baseline	Baseline	Baseline	0.11
	Sometimes	1.07 (0.78, 1.47)	1.26 (0.57, 2.79)	0.17 (0.05, 0.57)	1.14 (0.63, 2.04)	1.16 (0.67, 1.99)	
	Always	1.06 (0.75, 1.50)	0.97 (0.40, 2.31)	0.18 (0.05, 0.59)	0.97 (0.48, 1.96)	1.24 (0.69, 2.21)	
Risk taking	Avoid taking risks	Baseline	Baseline	Baseline	Baseline	Baseline	0.78
	Somewhere in between	0.95 (0.69, 1.29)	1.25 (0.50, 3.09)	1.33 (0.61, 2.89)	0.89 (0.50, 1.60)	1.59 (0.86, 2.92)	
	Take risks	1.17 (0.84, 1.64)	0.93 (0.49, 1.76)	1.32 (0.60, 2.89)	0.82 (0.39, 1.71)	0.89 (0.54, 1.49)	
Perceived chance of HIV, next 3 months	No chance	Baseline	Baseline	Baseline	Baseline	Baseline	0.72
	Some chance	0.77 (0.58, 1.01)	0.85 (0.45, 1.62)	0.79 (0.41, 1.52)	0.58 (0.35, 0.95)	0.75 (0.45, 1.24)	
	Moderate chance	0.64 (0.39, 1.03)	0.82 (0.32, 2.12)	0.63 (0.21, 1.93)	0.49 (0.18, 1.29)	0.55 (0.22, 1.35)	
	High chance	0.50 (0.21, 1.20)	0.26 (0.03, 2.57)	1.33 (0.29, 6.01)	0.63 (0.06, 6.25)	0.08 (0.01, 0.72)	
Depression	No	Baseline	Baseline	Baseline	Baseline	Baseline	0.97
	Yes	0.84 (0.61, 1.17)	0.93 (0.50, 1.74)	0.93 (0.47, 1.83)	0.67 (0.17, 2.66)	0.86 (0.51, 1.43)	
Anxiety	No	Baseline	Baseline	Baseline	Baseline	Baseline	0.30
	Yes	1.05 (0.75, 1.47)	1.07 (0.57, 1.99)	1.73 (0.85, 3.54)	0.89 (0.24, 3.37)	0.79 (0.46, 1.37)	
PTSD symptoms	No	Baseline	Baseline	Baseline	Baseline	Baseline	0.20
	Yes	0.78 (0.58, 1.05)	0.67 (0.34, 1.32)	0.50 (0.24, 1.01)	1.15 (0.64, 2.06)	0.74 (0.42, 1.31)	
Binge drinking	Never	Baseline	Baseline	Baseline	Baseline	Baseline	0.06
	< Monthly	1.12 (0.77, 1.65)	1.15 (0.56, 2.39)	0.67 (0.31, 1.44)	1.04 (0.28, 3.85)	1.44 (0.71, 2.90)	
	Monthly	1.22 (0.81, 1.84)	0.60 (0.26, 1.38)	2.32 (0.96, 5.56)	0.76 (0.29, 2.03)	1.32 (0.58, 3.00)	
	≥ Weekly	1.04 (0.63, 1.70)	1.03 (0.39, 2.71)	0.66 (0.24, 1.79)	1.63 (0.36, 7.48)	1.44 (0.56, 3.72)	
Drug use past 30 days	No	Baseline	Baseline	Baseline	Baseline	Baseline	0.20
	Yes	1.11 (0.76, 1.63)	1.26 (0.60, 2.62)	1.01 (0.52, 1.97)	0.42 (0.13, 1.33)	1.66 (0.74, 3/72)	
Have heard of PrEP	No	Baseline	Baseline	Baseline	Baseline	Baseline	0.62
	Yes	1.00 (0.75, 1.33)	0.76 (0.41, 1.41)	0.87 (0.46, 1.65)	1.36 (0.69, 2.67)	1.01 (0.60, 1.67)	
Would disclosure PrEP to partner	No	Baseline	Baseline	Baseline	Baseline	Baseline	0.58
	Yes	0.71 (0.55, 0.92)	0.64 (0.33, 1.24)	0.73 (0.37, 1.42)	0.58 (0.36, 0.96)	0.87 (0.57, 1.34)	

^a^ Result of test for interaction to assess whether associations between characteristics and preference for on‐demand versus daily PrEP differed between settings.

### 4.2 Reasons for PrEP preferences

The commonest reasons for preferring on‐demand PrEP were: I don't like taking tablets every day (77%) and I am not at risk most of the time (55%). The commonest reasons for preferring daily PrEP were: daily PrEP provides protection all the time (76%) and daily PrEP gives more protection than on‐demand (65%) (Table 4).

**Table 4:** Reasons for PrEP preferences

Characteristic	Prefer on‐demand	Prefer daily
Easiest PrEP option		
Take two pills before sex and one after	314 (41%)	22 (4.2%)
Take two pills after you have sex	97 (13%)	16 (3.1%)
Take two pills before you have sex	339 (44%)	19 (3.6%)
Take a pill every day whether you are having sex or not	16 (2.1%)	464 (89%)
Pay for PrEP if same price as hot meal		
No	215 (28%)	164 (32%)
Yes	551 (72%)	357 (69%)

If prefer on‐demand PrEP, why?		
I don't like taking tablets everyday		
No	175 (23%)	0
Yes	591 (77%)	0
I am not at risk most of the time so would not need PrEP everyday		
No	343 (45%)	0
Yes	423 (55%)	0
Less tablets means less chance of getting side effects		
No	463 (60%)	0
Yes	303 (40%)	0
Taking PrEP everyday may make people think that I have HIV		
No	419 (55%)	0
Yes	347 (45%)	0
There will be less tablets than daily PrEP, so I will be able to store them more		
No	518 (68%)	0
Yes	248 (32%)	0
It would be cheaper than taking everyday		
No	457 (60%)	0
Yes	309 (40%)	0
Main reason for preferring on‐demand PrEP		
I don't like taking tablets everyday	300 (39%)	0
I am not at risk most of the time so would not need PrEP everyday	135 (18%)	0
Less tablets means less chance of getting side effects	77 (10%)	0
Taking PrEP everyday may make people think that I have HIV. On‐demand PrEP is different	117 (15%)	0
There will be less tablets than daily PrEP, so I will be able to store them more easily	30 (3.9%)	0
It would be cheaper than taking everyday	34 (4.4%)	0
Not sure	2 (0.3%)	0
Other	71 (9.3%)	0

If prefer on daily PrEP, why?		
I am at risk most of the time so I would need PrEP everyday		
No	0	373 (72%)
Yes	0	148 (28%)
Daily PrEP provides protection all the time so I don't need to plan when I have		
No	0	124 (24%)
Yes	0	397 (76%)
I think that daily PrEP gives more protection than on‐demand PrEP		
No	0	178 (34%)
Yes	0	343 (66%)
I like the routine of daily tablets rather than having to remember PrEP just at		
No	0	273 (52%)
Yes	0	248 (48%)
I do not plan sex; therefore, on‐demand PrEP would be difficult to take		
No	0	232 (45%)
Yes	0	289 (56%)
To reduce the chance of getting side effects		
No	0	414 (80%)
Yes	0	107 (21%)
Main reason for preferring daily PrEP		
I am at risk most of the time so I would need PrEP everyday	0	53 (10%)
Daily PrEP provides protection all the time so I don't need to plan when I have sex	0	208 (40%)
I think that daily PrEP gives more protection than on‐demand PrEP	0	115 (22%)
I like the routine of daily tablets rather than having to remember PrEP just at times of sex	0	55 (11%)
I do not plan sex; therefore, on‐demand PrEP would be difficult to take	0	66 (13%)
To reduce the chance of getting side effects	0	18 (3.5%)
Other	0	5 (1.0%)

Our data show that AYP in SSA tend to prefer on‐demand over daily PrEP options, with on‐demand most preferred in Uganda, among males and participants 18‐ to 24‐year‐olds. These data support research suggesting that on‐demand PrEP may be preferred among AYP as the infrequent dosing makes it less burdensome and more discreet [31]. The difficulty of adhering to a strict dosing regimen and predicting when sex will occur might deter AYP from on‐demand PrEP.

Overall, while there has been considerable research into PrEP preferences both before and after its availability, showing similar findings to our study, the settings were near exclusive to MSM in the Global North [20, 32–41]. Our study provides insight into settings with the most substantial burden of the HIV epidemic, among a uniquely vulnerable group and where healthcare implementation has significant challenges. Similar findings were observed among MSM in developed countries in Australia, France and the United States, where less frequent sex and being likely to anticipate when sex will occur were the main reasons to opt for on‐demand PrEP [42, 43, 44].

Within our sample, on‐demand PrEP was more popular among males than females. Two studies among MSM in the United States and France showed a high preference for on‐demand PrEP [45]. In contrast, in Montreal, Belgium and the Netherlands, daily PrEP was preferred among MSM [34, 35, 46]. A daily regimen seemed easier to incorporate into a daily routine and did not require planning for sex [47].

AYP aged 18‐ to 24‐year‐olds in our study were more likely to prefer on‐demand PrEP compared to 13‐ to 15‐year‐olds. This might be because with age and experience, as well as natural psychosocial development, AYP tend to start thinking more about the future as opposed to the “here and now,” and relationships become more stable making planning sexual encounters easier, allowing on‐demand PrEP to be a more viable option.

We found that participants who knew further in advance that they were likely to have sex, and have sex less frequently, were more likely to prefer on‐demand PrEP. This might be because these circumstances are more predictable and/or planned, therefore, demanding a less frequent HIV prevention regimen. This is supported by a US study showing that AYP assigned male at birth who were in favour of on‐demand PrEP were having sex infrequently [31]. We also observed that a sexual partner's known or unknown HIV‐positive status was associated with a preference for daily PrEP. This is likely due to the added security that taking PrEP on a daily set schedule could provide someone if they know their partner is HIV positive or are unsure of their status. Likewise, in our study, we observed participants who perceived having a greater risk of contracting HIV preferred daily PrEP, which may also reflect the added sense of security of a regular PrEP regimen. Participants willing to disclose their PrEP use to their partners were also more likely to prefer daily PrEP. This could be because those willing to tell their partner about their PrEP use are likely to prefer a more frequent regimen as they do not have to hide their PrEP use.

A new finding from our study was that participants having a larger age gap with their most recent partner were more likely to prefer daily PrEP. There is no existing literature on the relationship between partner age gap and PrEP preference, but an increased partner age gap is an established risk factor for HIV [48, 49]. Therefore, it might be likely that those engaging in sexual activity with older partners are aware of the added risk and uncertainty, and thus prefer a more routine PrEP regimen to minimize this risk. However, the extent to which partner age gap is correlated with HIV risk is far from clear [50, 51].

Our study has limitations. As a cross‐sectional study, we cannot ascertain causality for PrEP preference. Furthermore, we asked hypothetical questions about PrEP preference without actual PrEP usage. The data are self‐reported but may have response bias in those where the survey was interviewer‐administered. We did not use random sampling. The sampling approach does not allow generalizability, and we had limited power to assess associations separately within each country. Although participants received monetary reimbursement for their time, it is possible that this might have increased willingness to participate in the study.

## 5 CONCLUSIONS

Our data show that AYP in four SSA communities prefer on‐demand over daily PrEP options, with differences by site, age and sex. PrEP demand could be optimized and tailored to socio‐demographic differences and co‐designed with AYP.

## COMPETING INTERESTS

The authors declare no competing interests.

## AUTHORS’ CONTRIBUTIONS

JJD, NA, ELW, HAW and JF conceived and designed the manuscript. SH, GT, MM, LSC, TGN, ASS and RM participated in data collection. ELW conducted data analysis and assisted with interpretation. JJD and NA interpreted and wrote the original manuscript draft; all authors revised and approved the final version of the paper.

## FUNDING

The CHAPS study was funded by the European and Developing Countries Trial Partnership grant (EDCTP‐2) programme supported by the European Union (grant number RIA2016MC‐1616‐CHAPS). The work reported herein for Janan Janine Dietrich was made possible through funding by the South African Medical Research Council (SAMRC) through its Division of Research Capacity Development under the SAMRC Early Investigators Programme (for funding received from the South African National Treasury) as well as the CIPHER GROWING THE LEADERS OF TOMORROW grant from the International AIDS Society. Stefanie Hornschuh was supported by the Consortium for Advanced Research Training in Africa (CARTA). CARTA is jointly led by the African Population and Health Research Center and the University of the Witwatersrand and funded by the Carnegie Corporation of New York (Grant No. G‐19‐57145), Sida (Grant No:54100113), Uppsala Monitoring Center, Norwegian Agency for Development Cooperation (Norad), and by the Wellcome Trust [reference no. 107768/Z/15/Z] and the UK Foreign, Commonwealth & Development Office, with support from the Developing Excellence in Leadership, Training and Science in Africa (DELTAS Africa) programme.

## DISCLAIMER

The content hereof is the sole responsibility of the authors and does not necessarily represent the official views of the funders.

## Data Availability

The analysis dataset will be made available upon request and accessed through the LSHTM Data Compass repository (https://datacompass.lshtm.ac.uk/).
